# Professionalizing Gerontology: Why It is Essential to Credential the Education of Gerontologists

**DOI:** 10.1093/geroni/igaf122.2339

**Published:** 2025-12-31

**Authors:** Judith Sugar, Donna Schafer

**Affiliations:** University of Nevada, Reno, Reno, Nevada, United States; National Association for Professional Gerontologists, Healdsburg, California, United States

## Abstract

The National Association for Professional Gerontologists (NAPG) has credentialed the education and professional expertise of practicing gerontologists since 2007, in the absence of licensing in any U.S. state or Canadian province. To date, nearly 400 professionals have received a credential. Today it is more important than ever to credential gerontologists and professionalize gerontology. While many disciplines produce practitioners who support older adults and their families, it is graduates of gerontology degree programs who have the interdisciplinary breadth of knowledge, informed by a set of key gerontology competencies, to respond holistically to the multifaceted challenges experienced by older adults. First developed more than 30 years ago, the key gerontology competencies are periodically updated by GSA’s Academy of Gerontology in Higher Education (AGHE) and widely used by gerontology programs. Given the projected increase in the older population, the need for more trained gerontologists is apparent. However, the field has not kept pace with the need for qualified, trained gerontology practitioners by producing more graduates. As data presented in this poster indicate, there are no more gerontology degree programs today than there were 20 years ago. The poster also presents data derived from a survey of almost 100 credentialed NAPG members. Survey responses identify barriers to employability and career mobility, including an inadequate understanding on the part of employers about a trained gerontologist’s skills and competencies. The poster concludes with suggestions for enhancing the professional stature of credentialed gerontologists.

